# Neural mechanism of the relationship between sleep efficiency and clinical improvement in major depressive disorder: A longitudinal functional magnetic resonance imaging study

**DOI:** 10.3389/fpsyt.2022.1027141

**Published:** 2022-10-03

**Authors:** Tao Chen, Wenming Zhao, Yu Zhang, Jiakuai Yu, Ting Wang, Jiajia Zhang, Yifei Li, Jiajia Zhu, Dao-min Zhu

**Affiliations:** ^1^Department of Sleep Disorders, Affiliated Psychological Hospital of Anhui Medical University, Hefei, China; ^2^Hefei Fourth People’s Hospital, Hefei, China; ^3^Anhui Mental Health Center, Hefei, China; ^4^Department of Radiology, The First Affiliated Hospital of Anhui Medical University, Hefei, China

**Keywords:** major depressive disorder, functional magnetic resonance imaging, clinical symptoms, polysomnography, sleep efficiency

## Abstract

**Background:**

Antidepressants represent the most common treatment of choice for major depressive disorder (MDD). In this study, we aimed to explore the status-related changes (acute vs. remitted status) in brain function in patients with MDD.

**Methods:**

Regular antidepressant medications (an average of 7 months after the initial visit, remitted status) were received by 48 patients with MDD. All the patients underwent MRI and polysomnography examinations as well as clinical assessment at each visit.

**Results:**

We found that baseline fractional amplitude of low-frequency fluctuations (fALFF) of right superior parietal gyrus (SPG) and middle frontal gyrus could predict depression and anxiety symptoms improvement from acute to remitted status in patients with MDD, respectively. Moreover, we found a significant positive correlation between the fALFF of right SPG and baseline sleep efficiency (SE) in patients with MDD. Further mediation analysis revealed that the fALFF of right SPG mediated the relationship between baseline SE and depressive symptom improvement.

**Conclusion:**

Apart from highlighting the fALFF as a potential prognostic indicator to predict and track disease progression in patients with MDD, these findings might provide a neural mechanism basis for improving sleep quality of patients with MDD and thus promoting the recovery of clinical symptoms, as well as provide a practical basis for clinical interventions in patients with MDD with sleep disorders.

## Introduction

Major depressive disorder (MDD) is currently one of the leading causes of disability worldwide, with clinical manifestations of depressed mood, vegetative symptoms, cognitive decline, and sleep abnormalities (1, 2). Despite tremendous efforts, current understanding of the causes is limited, given its mixed findings about the effects of antidepressants treatment (3) and depression-related brain structure and function abnormalities (4) and complex symptomatology. Different individuals may have different neuropathological processes, or even the same individual may have different neuropathological processes at different times. Due to baseline neurobiological characteristics and their changes with treatment, more than half of patients with MDD recovered within 6 months, while a good proportion do not recover and go on to develop chronic depression (5–7). However, most of our understanding of the effects of antidepressants is at the receptor level, and the specific mechanisms by which antidepressant-induced biochemical alterations are translated into significant clinical effects are still poorly understood. Therefore, there is an urgent clinical need to find a marker of neurobiology that can more precisely predict longitudinal alterations in clinical symptoms in patients with MDD, in order to optimize treatment strategies and disease management.

Neuroimaging techniques provide a means to elucidate the brain dysfunction and possible mechanisms of antidepressant treatment in depression. As a non-invasive neuroimaging method, resting-state functional magnetic resonance imaging (rs-fMRI) has emerged to assess spontaneous brain activity based on the blood-oxygen-level-dependent (BOLD) signal (8). Amplitude of low-frequency fluctuations (ALFF) (9) and its normalized version fractional amplitude of low-frequency fluctuations (fALFF) (10) are the most commonly used metrics derived from rs-fMRI. They can measure the low-frequency oscillation intensity of BOLD time courses and reflect local neural activity strength. Moreover, fALFF has been widely used in the study of depression due to its ability to effectively suppress non-specific signal components and improve sensitivity and specificity compared with ALFF. It has achieved remarkable success in unraveling abnormalities of local neural activity in this condition (11–15), as well as investigated the antidepressant treatment neural effects and their relationships with improvement of clinical in a longitudinal manner (16–18). Despite these promising findings, however, the effect of antidepressant treatment on neural activity in depression is quite complex and needs to be further revealed.

Sleep disturbance is a common concomitant symptom of depression, while insomnia is the most common sleep disorder, including delayed sleep onset, frequent awakenings, early awakenings, and reduced sleep efficiency, affecting more than 90% of MDD patients (19–21). Many researches have been devoted to exploring the association of depression with sleep disturbance. In MDD, the genome-wide associations reported a strong genetic association of insomnia with depressive symptoms (22). And another study showed that insomnia was an independent predictor of depression severity and suicide risk (23). The presence of insomnia makes depression less responsive to treatment and increases the risk of depression episodes twofold to fourfold (24–26). And improve the insomnia of patient can also effectively alleviate the clinical symptoms (27, 28). Moreover, a lot of longitudinal studies have confirmed that insomnia is an independent risk factor for the new-onset or recurrent depression among young, middle-aged and older adults (29). Thus, sleep disturbance and depression are closely related and share a bidirectional relationship (29, 30).

Recently, a rapidly growing body of rs-MRI literature has emerged to explore the underlying neural mechanisms of various sleep disorders in depression. A study found that the severity of insomnia in patients with MDD was associated with the increase of ALFF in the right inferior frontal gyrus/anterior insula (31). Another study showed that the functional connectivity (FC) of the right perigenual anterior cingulate cortex to the left superior parietal gyrus significant mediates the correlation between sleep quality and anhedonia in patients with MDD, namely, lower sleep quality leads to the FC depletion that in turn induces more severe anhedonia (32). Nevertheless, these studies were based on subjective sleep quality assessments [i.e., the sleep items of the Pittsburgh Sleep Quality Index (PSQI) and Hamilton Rating Scale for Depression (HAMD)] rather than objective tools [e.g., polysomnography (PSG)]. It is the gold standard for measuring sleep and provides objective data on many sleep parameters, such as total time in bed (TIB), total sleep time (TST), sleep efficiency (SE), latency of sleep, non-rapid eye movement sleep duration (NREM), and rapid eye movement sleep duration (REM) (33). Thus, PSG provides objective sleep parameters for the study of brain function in patients with MDD (33, 34). Among these sleep parameters, SE not only takes into account all sleep continuous variables (latency of sleep onset, wake after sleep onset and sleep period time) in its calculations, but can well reflect the increase of TIB and the decrease of efficient, stable and uninterrupted sleep time caused by decreased energy in patients with MDD (33, 35, 36). Remarkably, our earlier neuroimaging research showed that the low SE group (SE < 90%) had lower fALFF in right middle temporal gyrus, cuneus and thalamus compared with the normal SE group (SE ≥ 90%) in patients with MDD, and the resting-state FC of right cuneus to right lateral temporal cortex significantly mediated the correlations between SE and clinical symptoms severity (15). Therefore, the neural mechanism of sleep disorders in MDD can be better captured by SE.

Herein, we sought to perform a longitudinal study to explore the potential association between brain neural activity, clinical symptoms improvement and sleep in patients with MDD. We hypothesized (1) patients with MDD would exhibit longitudinal fALFF, clinical symptoms and sleep alterations from acute to remitted status; (2) baseline fALFF could predict clinical symptoms improvement and sleep change, (3) longitudinal fALFF alterations would be related with clinical symptoms improvement and sleep change, (4) there may be a plausible association between fALFF, clinical symptoms improvement and sleep.

## Materials and methods

### Participants

Forty-eight patients with MDD were consecutively recruited from Affiliated Psychological Hospital of Anhui Medical University. According to the International Classification of Diseases (ICD-10) criteria, the MINI-International Neuropsychiatric Interview (M.I.N.I.) was used to confirm the diagnoses of MDD by two well-trained clinical psychiatrists. HC were carefully screened to confirm an absence of any psychiatric illness using the M.I.N.I. Inclusion criteria included (1) age: 18–65 years; (2) Han Chinese; (3) right-handedness. Exclusion criteria included (1) presence of other psychiatric disorders such as psychiatric bipolar disorder, classification disorder, organic mental disorder, and psychotic disorders due to substance abuse and addiction; (2) severe physical illness; (3) history of epilepsy, head trauma, or other causes of loss of consciousness for more than 5 min; (4) contraindications to MRI scanning; (5) pregnant or lactating women. Regular antidepressant medications were received by all patients, including serotonin norepinephrine reuptake inhibitors (SNRIs), selective serotonin reuptake inhibitors (SSRIs) or noradrenergic and specific serotonergic antidepressant (NaSSA). These patients completed 2 study visits: baseline (initial visit, acute status) and follow-up (an average of 7 months after the initial visit, remitted status). We choose this period as follow-up because it is the routine time frame for determining recovery from a depressive episode and is therefore suitable for assessing clinical and psychosocial outcomes. All patients underwent PSG and MRI examinations, as well as clinical assessment at each visit. The Ethics Society of The First Affiliated Hospital of Anhui Medical University approved this study. All patients voluntarily participated in this experiment and signed the informed consent form after determining that they had fully understood the relevant contents of this experiment.

### Demographic and clinical variables

The trained research staff collected the demographic and clinical data, including age, gender, education, illness duration, and antidepressants. The HAMD is currently the most commonly used scale in psychiatry to assess the presence and severity of depression. In this study, the 24-item HAMD scale was used to rate the depressive symptom of patients with MDD at baseline and follow-up, with a total score of 8–19 being mild depression; 20–34 being moderate depression and ≥ 35 being severe depression (37). At each visit, the anxiety symptom of patients with MDD was assessed by the Hamilton Rating Scale for Anxiety (HAMA). A total score of more than 29 points may indicate severe anxiety; over 21 points, there must be significant anxiety; anything over 14 points is definitely anxiety; more than 7 points, probably anxiety; if less than 7 points, no anxiety (38).

Subjective sleep quality was assessed by using the PSQI, which is used to rate the quality of sleep in the last 1 month. The scale was divided into seven dimensions: subjective sleep quality, latency of sleep, duration of sleep, habitual sleep efficiency, sleep disturbances, use of sleeping medication, and daytime dysfunction. Each dimension is scored according to 0–3 points, and the accumulated score of each component is the PSQI total score, which ranges from 0 to 21 points. The higher the score is, the worse the sleep quality is (39).

### Polysomnography examination

On an Embla N7000 (New York, USA) instrument, all patients with MDD underwent overnight PSG examinations at each visit. Cardiopulmonary parameters (electrocardiogram, chest and abdominal movements, oxygen saturation, oral, and nasal flow) and neurophysiological parameter (electroencephalogram, electrooculography, chin electromyogram, and lower limb movements) were recorded automatically and subsequently analyzed manually. In this study, we extracted the following PSG variables: TST, TIB, and SE. TIB was defined as the time from “lights out” to “lights on,” while TST was defined as the sum of sleep time in NREM and REM sleep phases. SE can be obtained by the ratio of TST to TIB. As a comprehensive indicator of sleep quality, our subsequent analysis of PSG variables focused on it.

### Image acquisition

MRI scan were obtained using a 3.0-Tesla MR system (Discovery MR750w, General Electric, Milwaukee, WI, USA) with a 24-channel head coil. Earplugs were used to reduce scanner noise, and tight but comfortable foam padding was used to minimize head motion. During the scans, all participants were instructed to keep their eyes closed, relax but not fall asleep, think of nothing in particular, and move as little as possible. High-resolution 3D T1-weighted structural images were acquired by employing a brain volume (BRAVO) sequence with the following parameters: repetition time (TR) = 8.5 ms; echo time (TE) = 3.2 ms; inversion time (TI) = 450 ms; flip angle (FA) = 12°; field of view (FOV) = 256 mm × 256 mm; matrix = 256 × 256; slice thickness = 1 mm, no gap; 188 sagittal slices; and acquisition time = 296 s. Resting-state BOLD data were acquired using a gradient-echo single-shot echo planar imaging (GRE-SS-EPI) sequence with the following parameters: TR = 2,000 ms; TE = 30 ms; FA = 90°; FOV = 220 mm × 220 mm; matrix = 64 × 64; slice thickness = 3 mm, slice gap = 1 mm; 35 interleaved axial slices; 185 volumes; and acquisition time = 370 s. Routine T2-weighted images were also collected to exclude any organic brain abnormality. All images were visually inspected to ensure that only images without visible artifacts (e.g., ghosting artifacts arising from subject movement and pulsating large arteries, metal artifacts, susceptibility artifacts, blooming artifacts) were included in subsequent analyses. None of the participants was excluded for visually inspected imaging artifacts.

### Functional magnetic resonance imaging data pre-processing and fractional amplitude of low-frequency fluctuations analysis

The Data Processing and Analysis for Brain Imaging (DPABI)^1^ (40) and Statistical Parametric Mapping software (SPM12)^2^ were used to preprocess the rs-fMRI data. For each subject, the first 10 volumes were discarded to allow the signal to reach equilibrium and the participants to adapt to the scanning noise. The remaining volumes were corrected for the acquisition time delay between slices. To correct the motion between time points, realignment was performed. By estimating the translation in each direction and the angular rotation on each axis for each volume, we computed the head motion parameters. All participants’ BOLD data were within the defined motion thresholds (i.e., maximum translational or rotational motion parameters less than 2 mm or 2°). We also calculated FD, which indexes the volume-to-volume changes in head position. Several nuisance covariates (the cerebrospinal fluid signal, the white matter signal, the estimated motion parameters based on the Friston-24 model, the linear drift, and the spike volumes with FD > 0.5) were regressed out from the data. Individual structural images were firstly co-registered with the mean functional image in the normalization step; then the transformed structural images were segmented and normalized to the Montreal Neurological Institute (MNI) space using the diffeomorphic anatomical registration through the exponentiated Lie algebra (DARTEL) technique (41). Lastly, each filtered functional volume was spatially normalized to MNI space using the deformation parameters estimated during the above step and resampled into a 3 mm isotropic voxel. After spatial normalization, all data sets were smoothed with a 6 mm full-width at half-maximum (FWHM) Gaussian kernel.

The BOLD time course of each voxel obtained from the preprocessed data was transformed to a frequency domain *via* a Fast Fourier Transform (FFT). Then the power spectrum was obtained. fALFF was defined as the ratio of the power spectrum in the low-frequency band (0.01–0.1 Hz) to that in the entire frequency range (10). For the purpose of standardization, the fALFF value of each voxel was divided by the global mean fALFF value, yielding a standardized fALFF map per subject.

### Statistical analyses

The paired *t*-test was used to test the changes in clinical symptoms (i.e., HAMD and HAMA scores) and sleep (i.e., PSQI scores and PSG variables) from baseline to follow-up in all MDD patients. The SPSS 23.0 software package (SPSS, Chicago, IL, USA) was used to perform these statistical analyses. The significant threshold was set at *P* < 0.05 (two-tailed).

Our fALFF maps data analytic strategy comprised 3 steps. First, paired *t*-tests were used to examine fALFF alteration from baseline to follow-up in patients with MDD. Next, if a clinical manifestation was significant change from baseline to follow-up, we test whether baseline fALFF could predict this clinical manifestation change. Thus, voxel-wise correlation of baseline fALFF with the change in this clinical manifestation from baseline to follow-up was performed using multiple regression analyses controlling for age, gender, education, and FD. Multiple comparison correction was performed using the cluster-level family-wise error (FWE) method for the voxel-based analyses, resulting in a cluster defining threshold of *P* = 0.001 and a corrected cluster significance of *P* < 0.05. The fALFF value of each cluster with significant correlation with the change in this clinical manifestation for each subject was extracted and used for subsequent region of interest (ROI)-based analyses with other clinical variables. For completeness, if significant association of baseline fALFF with the change in a clinical manifestation was found, we also calculated fALFF alterations (follow-up—baseline) within the significant brain regions, and their correlations with clinical change were then further tested. Finally, bootstrapped mediation analysis of a linear regression model was performed to test whether the relationship between variables was mediated by other variables, using the PROCESS macro^3^ (42, 43), a versatile modeling tool freely available for SPSS. In mediation analyses, only variables that showed a significant association with others were considered dependent, independent, or mediating variables. Age, gender, education and FD were included as covariates. Based on 5,000 boot-strap realizations, the bootstrap 95% confidence interval (CI) assessed the significance of mediation effects in the way a significant indirect effect is indicated when the bootstrap 95% CI does not include zero.

### Sensitivity analysis

We conducted the following procedures to further evaluate the reproducibility of our findings. To test the possible effects of illness duration and antidepressants on our results, we added these two factors as additional nuisance covariates in the above analyses, with illness duration as a continuous variable and antidepressants types (SSRIs, SNRIs, and NaSSA) as a categorical variable.

## Results

### Demographic, clinical and polysomnography characteristics

Table 1 lists the detailed demographic, clinical, and PSG data for sample. In brief, patients with MDD exhibited significant reductions in HAMD (*t* = –16.054, *P* < 0.001), HAMA (*t* = –15.765, *P* < 0.001), PSQI (*t* = –4.644, *P* < 0.001), TIB (*t* = –4.913, *P* < 0.001) and TST (*t* = –3.033, *P* < 0.001) from baseline to follow-up. However, there was no significant change in SE (*t* = 1.236, *P* = 0.220) from baseline to follow-up. Among the 48 patients, 36 received SSRIs, 9 SNRIs, and 3 NaSSA.

**TABLE 1:** Demographic, clinical, and PSG characteristics of the sample.

Characteristics	Baseline	Follow-up	Statistics	*P*-value
Age (yeas)	44.50 ± 10.08	–	–	–
Gender (female/male)	33/15	–	–	–
Education (years)	8.80 ± 3.30	–	–	–
Illness duration (months)	67.02 ± 82.30	–	–	–
HAMD	31.70 ± 11.20	5.70 ± 3.70	*t* = –16.054	<0.001
HAMA	21.20 ± 7.70	3.90 ± 2.40	*t* = –15.765	<0.001
PSQI	13.30 ± 4.90	9.70 ± 4.30	*t* = –4.644	<0.001
FD (mm)	0.13 ± 0.09	0.16 ± 0.11	*t* = 1.834	0.070
**PSG data**				
TIB (min)	516.90 ± 49.70	464.90 ± 58.60	*t* = –4.913	<0.001
TST (min)	445.00 ± 60.60	410.80 ± 57.80	*t* = –3.033	0.004
SE	0.86 ± 0.10	0.88 ± 0.07	*t* = 1.236	0.220
**Antidepressant medications (numbers of patients)**				
SSRIs	39	–	–	–
SNRIs	9	–	–	–
NaSSA	3	–	–	–

PSG, polysomnography; HAMD, Hamilton Rating Scale for Depression; HAMA, Hamilton Rating Scale for Anxiety; PSQI, Pittsburgh Sleep Quality Index; FD, frame-wise displacement; TIB, total time in bed; TST, total sleep time; SE, sleep efficiency; SSRIs, selective serotonin reuptake inhibitors; SNRIs, serotonin norepinephrine reuptake inhibitors; NaSSA, noradrenergic and specific serotonergic antidepressant.

### Longitudinal change in fractional amplitude of low-frequency fluctuations analysis

The alteration in fALFF from baseline to follow-up did not survive multiple comparisons correction in patients with MDD (*P* > 0.05, cluster-level FWE corrected).

### Associations between fractional amplitude of low-frequency fluctuations analysis and clinical improvement

**FIGURE 1:**
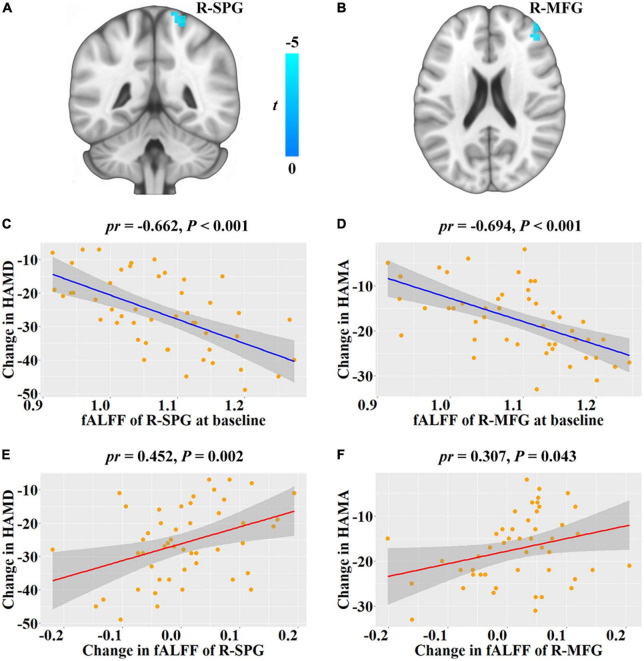
Correlations between fALFF and depressive and anxiety symptoms changes from baseline to follow-up in patients with major depressive disorder. **(A,B)** Brain regions show significant correlations between baseline fALFF and HAMD and HAMA changes, respectively. The cold color denotes the negative correlations. **(C,D)** Scatter plots of the correlations between baseline fALFF of the significant clusters and HAMD and HAMA changes, respectively. **(E,F)** Scatter plots of the correlations between fALFF change of the significant clusters and HAMD and HAMA changes, respectively. Change = follow-up—baseline. fALFF, fractional low frequency amplitude; HAMD, Hamilton Rating Scale for Depression; HAMA, Hamilton Rating Scale for Anxiety; R, right; SPG, superior parietal gyrus; MFG, middle frontal gyrus; *pr*, partial correlation coefficient.

Since significant improvement in clinical symptoms and subjective sleep in post-treatment, we tested the correlations of baseline fALFF with the changes of them. Then, we found that baseline fALFF in the right superior parietal gyrus (SPG, cluster size = 28 voxels, peak MNI coordinate x/y/z = 15/–51/69, peak *t* = 4.971, Figure 1A) was negative association with HAMD change from baseline to follow-up, and baseline fALFF in the right middle frontal gyrus (MFG, cluster size = 29 voxels, peak MNI coordinate x/y/z = 42/54/24, peak *t* = –4.708, Figure 1B) was negative association with HAMA change from baseline to follow-up (*P* < 0.05, cluster-level FWE corrected), i.e., patients with higher baseline fALFF demonstrated a greater depression and anxiety symptoms improvement (Figures 1C,D). Further analysis certified that the fALFF alterations in right SPG and MFG from baseline to follow-up were positive associations with HAMD and HAMA changes, respectively, i.e., patients with greater reduction in fALFF verified greater depression and anxiety symptoms improvement (Figures 1E,F). However, we did not find any significant association of the PSQI change from baseline to follow-up with baseline fALFF in patients with MDD (*P* > 0.05, cluster-level FWE corrected).

**FIGURE 2:**
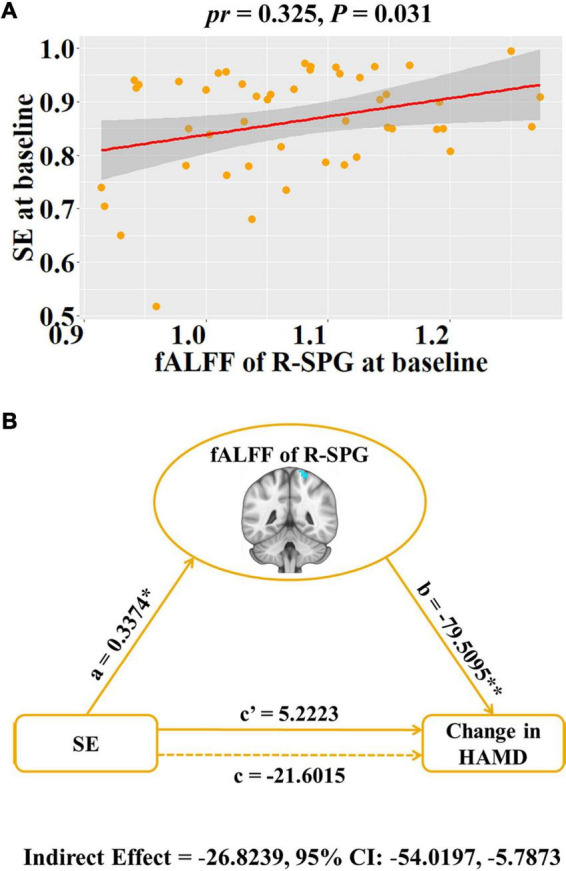
Associations between baseline fALFF, baseline SE and HAMD change in patients with major depressive disorder. **(A)** Scatter plots of the correlation between baseline SE and the fALFF of R-SPG. **(B)** Graphical representation of the mediation analysis between baseline SE and HAMD change with the fALFF of R-SPG as the mediator: estimates of the mediated (a × b), direct (c’), and total (c) effects. Change = follow-up—baseline. *P < 0.05, **P < 0.001. fALFF, fractional low frequency amplitude; SE, sleep efficiency; HAMD, Hamilton Rating Scale for Depression; R, right; SPG, superior parietal gyrus; *pr*, partial correlation coefficient; CI, confidence interval.

For ROI analyses, we found that baseline SE was significant positive association with fALFF of right SPG (*pr* = 0.325, *P* = 0.031, Figure 2A), but not fALFF of R-MFG (*P* > 0.05). Moreover, there was no significant correlation of baseline PSQI with fALFF of right SPG or MFG, and no significant correlation of PSQI change with HAMD or HAMA changes (*P* > 0.05).

### Mediation analysis

To explore the potential association among baseline SE, the fALFF of right SPG and depressive symptom improvement (i.e., HAMD change), the SE-fALFF-HAMD change mediation model was tested where the fALFF of right SPG mediated the relationship between baseline SE and HAMD change. We found that the relationship between baseline SE and HAMD change was significantly mediated by the fALFF of the right SPG (indirect effect = –26.8239, standard error = 11.8584, 95% CI: –54.0197, –5.7873, Figure 2B).

### Sensitivity analysis

After additionally controlling for illness duration and antidepressant types, the associations between the fALFF of right SPG and HAMD change (*pr* = –0.676, *P* < 0.001), the fALFF of right MFG and HAMA change (*pr* = –0.698, *P* < 0.001), the fALFF alteration of right SPG and HAMD change (*pr* = 0.457, *P* = 0.002), the fALFF alteration of right MFG and HAMA change (*pr* = 0.305, *P* = 0.045), and baseline SE and the fALFF of SPG (*pr* = 0.319, *P* = 0.040) were preserved. Moreover, the mediation result was also reproducible (indirect effect = –27.3356, standard error = 12.2158, 95% CI: –58.3397, –7.3942).

## Discussion

In this study, we investigated longitudinal fALFF alteration and clinical manifestations changes from acute to remitted status, as well as the correlations between fALFF, clinical symptoms improvement and sleep in MDD patients. Contrary to our expectations, we found no longitudinal fALFF alteration and objective sleep (i.e., SE) change from acute to remitted status in MDD patients. However, we observed the significant improvement in depression and anxiety symptoms as well as subjective sleep (i.e., PSQI), and baseline fALFF of right SPG and MFG could predict depression and anxiety symptoms improvement from acute to remitted status, respectively. Furthermore, longitudinal fALFF alterations in these regions were correlated to the degree of symptoms improvement. In addition, the fALFF of right SPG was positive correlation with baseline SE in MDD patients. Further mediation analysis found that the fALFF of right SPG mediated the relation between baseline SE and HAMD change in patients with MDD.

In agreement with previous studies (44–48), we found that depression and anxiety symptoms as well as subjective sleep (PSQI scores) were significant improvement in follow-up. However, we did not find any longitudinal fALFF alteration from acute to remitted status in MDD patients, which was inconsistent with several previous studies (16–18). This discrepancy may be explained by differences in illness profiles (chronic vs. first-episode MDD), therapies (antidepressant vs. combined therapy). Moreover, among the PSG parameters, the significant reduction in TIB of patients indicates a significant improvement in excessive bed time due to energy loss caused by the disease, while the reduction in TST may be due to the reduction in total bed time of patients (35). We acknowledge that patients had poor SE at baseline, but there was no statistically significant improvement after treatment. This may be due to improved mood and awareness of sleep after treatment, leading to a reduction in unnecessary bed time.

Moreover, we found that higher baseline fALFF of right SPG and MFG could predict greater improvement in depression and anxiety symptoms in patients with MDD, with a greater fALFF reduction corresponding to greater symptoms improvement. The SPG, as an important part of the dorsal attention network (DAN), is associated with top-down, goal-driven attention, memory extraction, and spatial task processing (49, 50). Alterations in neural function of the SPG may be associated with abnormalities in DAN function and cognitive abilities such as attention, learning, and memory in patients with MDD (51). A recent longitudinal study found that patients with sub threshold depression can reduce depressive symptoms by enhancing the ALFF value of the right SPG through physical exercise (52). The MFG, which has been recognized as an important brain region for attentional processing, is a region of the ventral lateral prefrontal cortex that connects the DAN and the ventral attention network (VAN) (53). In addition, there is growing convergent evidence that the neural activity of MFG has emerged as a promising prognostic marker of MDD treatment outcome (16–18), which is consistent with our current finding. These data invite us to speculate that decreased neural activity of MFG confers better treatment outcomes by helping to recalibrate interactions between the DAN and VAN.

Previous brain imaging studies of MDD patients with sleep disorders have mostly focused on the amygdala, orbitofrontal cortex, and cingulate cortex, but rarely on parietal regions (54–56). A recent study of 1,053 MDD patients with insomnia found that the more severe the insomnia, the smaller the cortical surface area of several brain regions, including the right SPG (57). In this study, there was a significant positive correlation between baseline SE and local neural activity in right SPG. And more importantly, the fALFF of right SPG was found to play an important mediator role between baseline SE and improvement in depressive symptom. We speculate that this is because the sleep problems of MDD patients cause the defect of attention function, which leads to the abnormal DAN function including the SPG, thus affecting the improvement of patients’ clinical symptoms. On the one hand, although the relationship is complex, our findings provide preliminary evidence that the effects of SE on depressive symptom appear to have a neuroanatomical basis. On the other hand, these results provide a practical basis for clinical interventions in MDD patients with sleep disorders.

There are several limitations that need to be mentioned. First, the healthy controls were not recruited in our study. Since the known as the “first night effect,” significant changes in sleep patterns between the first and second nights in the hospital will always challenge one night PSG (58). Moreover, this effect was more pronounced in healthy controls than in patients who spent several days in a hospital to acclimatize to their sleep environment. A study has shown that depression in-patients have relatively little sleep adaptations change (59). Therefore, first-night PSG data from in-patients are relatively reliable, whereas incorrect first-night PSG data from healthy controls may result in unreliable outcomes. This issue may be solved by other sleep measurement methods such as actigraphy in the future. Second, confounding factors such as illness duration and/or drug use may influence the current findings. Remarkably, the outcomes of correlation analyses were remained after controlling for illness duration and antidepressant types. Nevertheless, in future studies, our findings should be validated using first-line medication naive MDD patients. Third, although we adopted several steps to ensure that the participants did not fall asleep during the scanning, we cannot exclude its effects completely. Fourth, HAMD scores are usually noisy and more refined assessment of depressive symptoms would likely add value to future studies. Finally, the statistical power in detecting subtle brain changes and uncovering potential relationship of depression-brain-sleep may be limited due to the fairly modest sample size. Larger sample is needed to validate our results in future study.

In conclusion, our data suggest that the fALFF has the potential to serve as early biomarker of clinical improvement and longitudinal fALFF alteration can be used to track the disease progression in MDD patients. More importantly, our data revealed a plausible relationship between brain neural activity, baseline SE, and depressive symptom improvement in MDD patients. Our findings not only provide a neural mechanism basis for improving the sleep quality of MDD patients and thus promoting the recovery of clinical symptoms, but also provide a practical basis for clinical interventions in MDD patients with sleep disorders.

## Data availability statement

The original contributions presented in this study are included in the article/supplementary material, further inquiries can be directed to the corresponding author/s.

## Ethics statement

The studies involving human participants were reviewed and approved by the Ethics Committee of The First Affiliated Hospital of Anhui Medical University. The patients/participants provided their written informed consent to participate in this study.

## Author contributions

D-MZ and JZhu conceptualized and designed the study. TC and WZ were responsible for conducting the analyses, preparing the first draft of the manuscript, and preparing the manuscript for submission. D-MZ was responsible for obtaining funding for the study, supervising the analyses, and editing drafts of the manuscript. YZ, JY, TW, JZha, and YL were responsible for data collection and initial data preprocessing. All authors contributed to and approved the final manuscript.

## Footnotes

1
http://rfmri.org/dpabi

2
http://www.fil.ion.ucl.ac.uk/spm

3
http://www.processmacro.org/
